# Comprehensive validation of T- and B-cell deficiency in *rag1*-null zebrafish: Implication for the robust innate defense mechanisms of teleosts

**DOI:** 10.1038/s41598-017-08000-2

**Published:** 2017-08-08

**Authors:** Yumie Tokunaga, Masamichi Shirouzu, Ryota Sugahara, Yasutoshi Yoshiura, Ikunari Kiryu, Mitsuru Ototake, Takahiro Nagasawa, Tomonori Somamoto, Miki Nakao

**Affiliations:** 1 Laboratory of Marine Biochemistry, Department of Bioscience and Biotechnology, Graduate School of Bioresource and Bioenvironmental Sciences, Kyushu University, Fukuoka, 812-8581 Japan; 2 Yashima station, Stock Enhancement and Management Department, National Research Institute of Fisheries and Enhancement of Inland Sea, Japan Fisheries Research and Education Agency, 243 Yashima-higashi, Takamatsu, Kagawa 761-0111 Japan; 3 National Research Institute of Aquaculture, Fisheries Research Agency, Tamaki, Mie 519-0423 Japan; 4 Instituto de Medicina Molecular, Faculdade de Medicina, Universidade de Lisboa, Lisboa, Portugal

## Abstract

*rag1* ^−/−^ zebrafish have been employed in immunological research as a useful immunodeficient vertebrate model, but with only fragmentary evidence for the lack of functional adaptive immunity. *rag1*-null zebrafish exhibit differences from their human and murine counterparts in that they can be maintained without any specific pathogen-free conditions. To define the immunodeficient status of *rag1* ^−/−^ zebrafish, we obtained further functional evidence on T- and B-cell deficiency in the fish at the protein, cellular, and organism levels. Our developed microscale assays provided evidence that *rag1* ^−/−^ fish do not possess serum IgM protein, that they do not achieve specific protection even after vaccination, and that they cannot induce antigen-specific CTL activity. The mortality rate in non-vaccinated fish suggests that *rag1* ^−/−^ fish possess innate protection equivalent to that of *rag1* ^+/−^ fish. Furthermore, poly(I:C)-induced immune responses revealed that the organ that controls anti-viral immunity is shifted from the spleen to the hepatopancreas due to the absence of T- and B-cell function, implying that immune homeostasis may change to an underside mode in rag-null fish. These findings suggest that the teleost relies heavily on innate immunity. Thus, this model could better highlight innate immunity in animals that lack adaptive immunity than mouse models.

## Introduction

Recombination-activating gene 1 (*rag1*) plays an essential role in the rearrangement and recombination of immunoglobulins and T-cell receptor (TCR) genes. Rag1-deficient animals lack functional T- and B-cells in adaptive immunity, and thus, it is possible to investigate innate immunity that is not affected by T- and B-cells in this model^1–3^. As Rag deficiency results in severe combined immunodeficiency (SCID) in mice and humans, they must be maintained in specific-pathogen free (SPF) conditions. Thus, mammals are thought to depend heavily on adaptive immunity.

Teleost fish are primitive vertebrates with an adaptive immune system, and they possess T- and B-cell functionality equivalent to that in mammals^4–9^. Rag genes have been identified in many fish species^10–14^, and it has been confirmed that the antigenic diversity is attributable to the TCRs and immunoglobulins of teleost fish^8, 15^. In 2002, *rag1*-deficient zebrafish were established using the targeting induced local lesions in genomes method (TILLING)^16^. Thus far, several groups have utilized this fish for immunological studies and demonstrated their functions in some pathogenic models^17–19^. The observations in these studies and our findings suggest that the *rag1*-null zebrafish possess more robust *rag1*-independent immunity than mammalian models. For instance, this fish never develops SCID, and it can be maintained for a long period (at least 2 years) under conventional conditions (non-SPF environment)^16^ (our unpublished information). Furthermore, they display antigen-specific protection against intracellular bacterial infection, implicating that they have immunological memory^17^. Therefore, we examined whether the *rag1*-null fish completely lacks functional T- and B-cells.

Previous studies reported evidence of T- and B-cell deficiency in *rag1*-null fish. The lymphocyte population in the deficient animals was significantly smaller than that in wild-type fish^20^. In addition, the V segments of TCR and immunoglobulin mRNA were not found in the null animals. Although these findings indicate that the *rag1*-null fish exhibits one of the typical phenotypes of *rag1*-deficient animals, it remains unclear whether they actually lack the ability to produce immunoglobulin, whether they are protected by vaccination, and whether they exhibit specific cell-mediated immunity, such as cytotoxic T cell function. Thus, additional inspections at the protein, cellular, tissue, and organism levels are necessary to confirm immunodeficiency in the fish strain. The present study sought to establish assays for analyzing these questions at the protein and cellular levels for small fish, validating T- and B-cell deficiency, investigating the expression profiles of cytokines in *rag1*-null fish, and discussing differences in the balance of adaptive and innate immunity between mammals and teleosts.

## Results

### Detection of IgM protein in serum and IgM-positive cells in kidneys and blood

**Figure 1:**
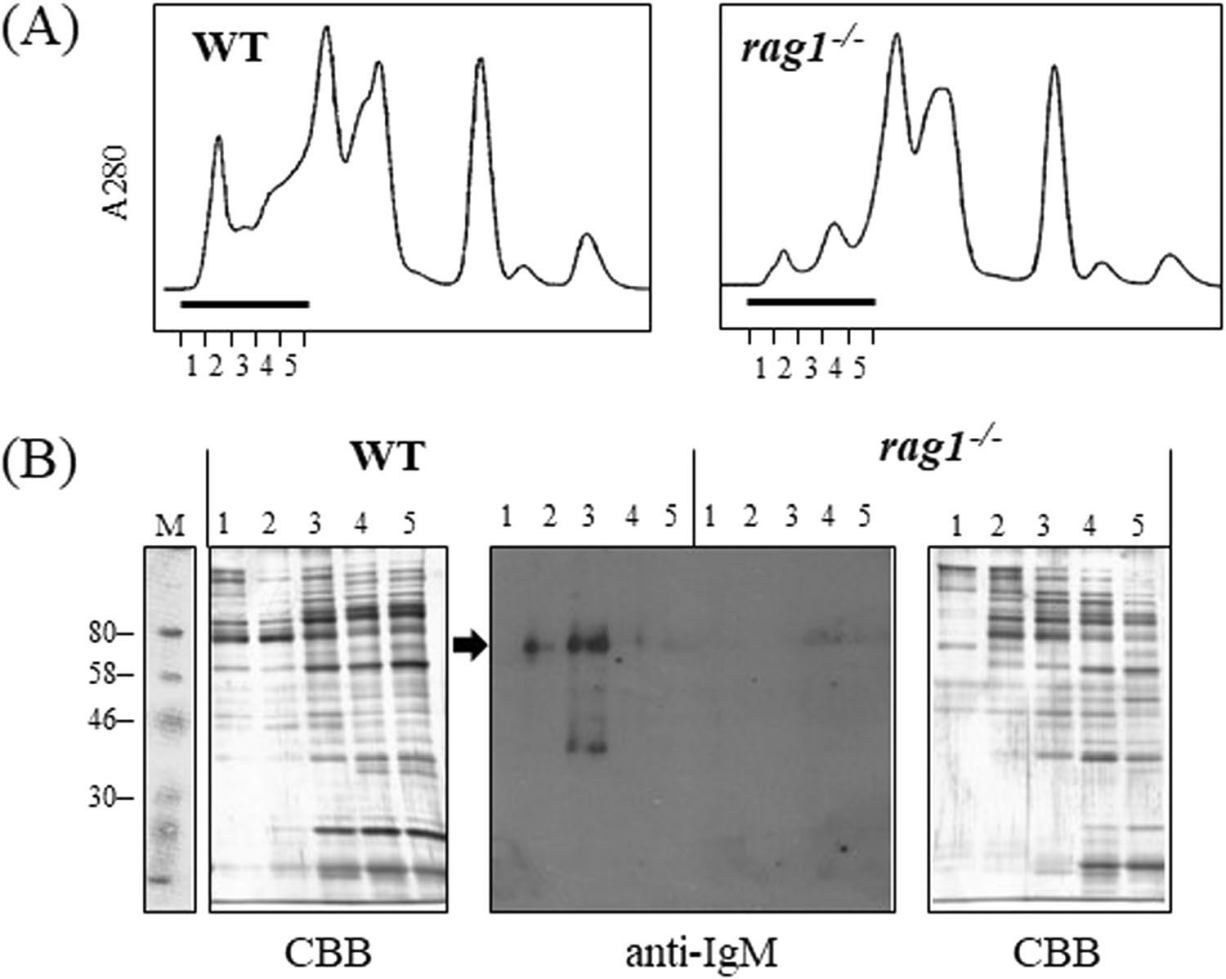
Validations of absence of IgM protein in serum. (**A**) Elution profiles in gel filtration chromatography of serum from wild-type (left) and *rag1* ^−/−^ zebrafish (right). Bars indicate the fractions that were applied to western blotting. Fraction numbers are indicated on the X-axis. (**B**) Detection of IgM in serum from wild-type (left) and *rag1* ^−/−^ fish (right) by western blotting. The arrow denotes the heavy chain of IgM (85 kDa). The numbers on the lane correlate to the fraction numbers in Fig. 1A.

Sera from *rag1* ^−/−^ and wild-type zebrafish were fractionated by gel filtration and analyzed for IgM expression by western blotting with chemiluminescent detection. The elution profiles exhibited marked differences in the high-molecular-weight fractions corresponding to tetrameric IgM, with lower protein peaks in *rag* ^−/−^ fish than in wild-type fish (Fig. 1A). In Western blotting of high-molecular-weight fractions (#1–5), the 85-kDa band of IgM heavy chain, which was evident in wild-type fish, was undetectable even by chemiluminescent detection in *rag1* ^−/−^ fish (Fig. 1B). These results indicate that IgM is absent in serum from *rag1* ^−/−^ fish.

**Figure 2:**
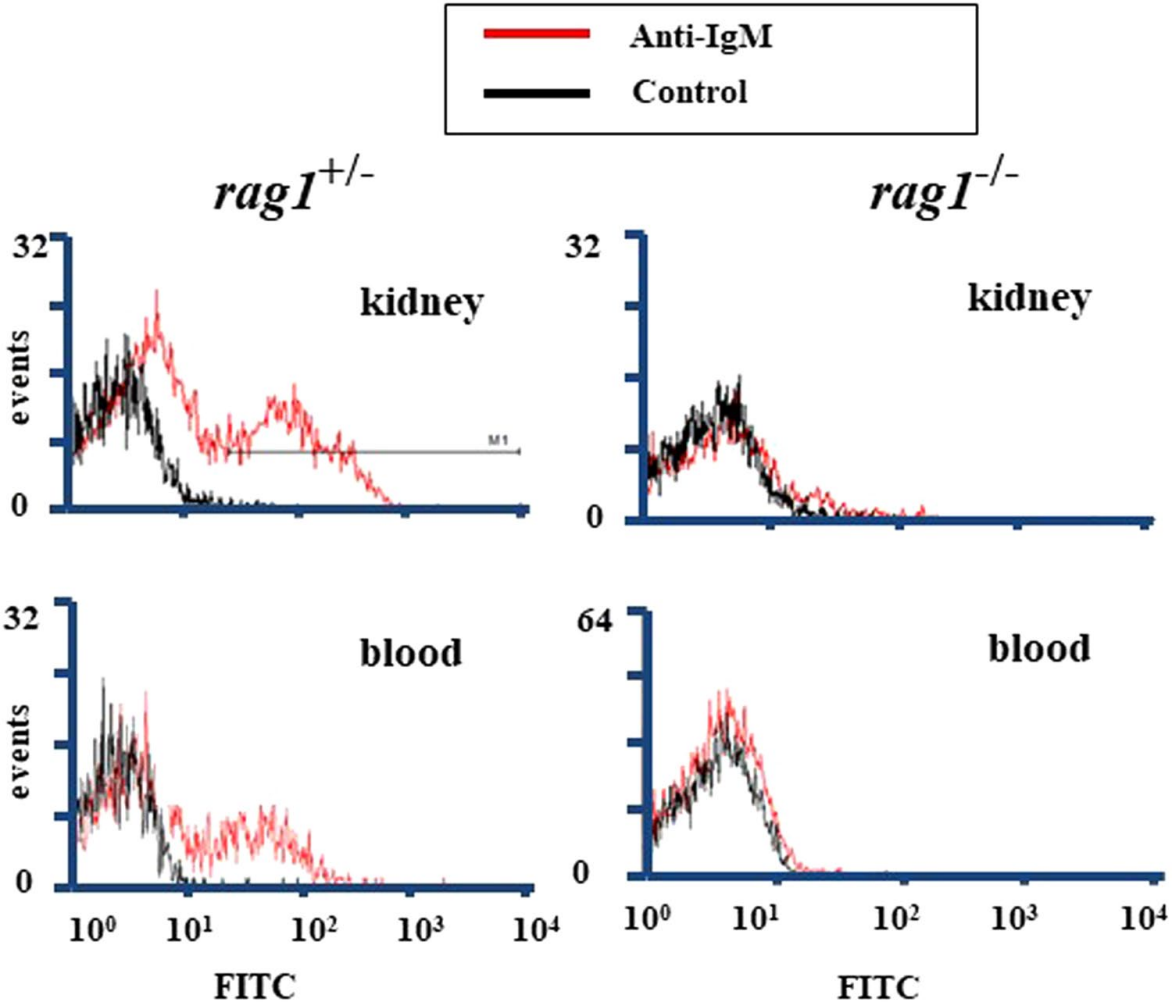
Flow cytometry analysis of IgM-positive leukocytes in kidneys and blood from *rag1* ^+/−^ and *rag1* ^−/−^ fish. Gray and black lines show anti-IgM–positive and anti-IgG–negative controls. Three individual samples were independently analyzed, and representative data are shown.

Membrane IgM (mIgM)-positive cells were detected in the kidneys and blood of *rag* ^+/−^ fish (28.3% and 15.5%, respectively). On the contrary, leukocytes in kidneys and blood from *rag* ^−/−^ fish contained less than 1.0% mIgM-positive cells, indicating that *rag* ^−/−^ fish lack mature IgM-positive B-cells (Fig. 2).

### Detection of antibody titers in vaccinated fish

To examine specific antibody production, the agglutinating antibody titer of serum from vaccinated fish was investigated in *rag1* ^+/−^ and *rag1* ^−/−^ animals. All sera from vaccinated *rag1* ^+/−^ fish displayed antibody titers of 32–320, whereas those of *rag1* ^−/−^ fish were below the detection limit (titer <16) (Table 1). The results clearly illustrate that *rag1* ^−/−^ fish completely lack specific antibody production.

**Table 1:** Antibody titer against vibrio in rag^−/−^ and rag^+/−^ zebrafish.

Fish No.	RAG1 genotype	Antibody titer
1	+/−	320
2	+/−	256
3	+/−	40
4	+/−	64
5	+/−	32
6	+/−	32
7	−/−	ND*
8	−/−	ND
9	−/−	ND
10	−/−	ND
11	−/−	ND
12	−/−	ND

*Antibody titers were less than 16.

### Protective effect of vaccination following infection with bacteria

**Figure 3:**
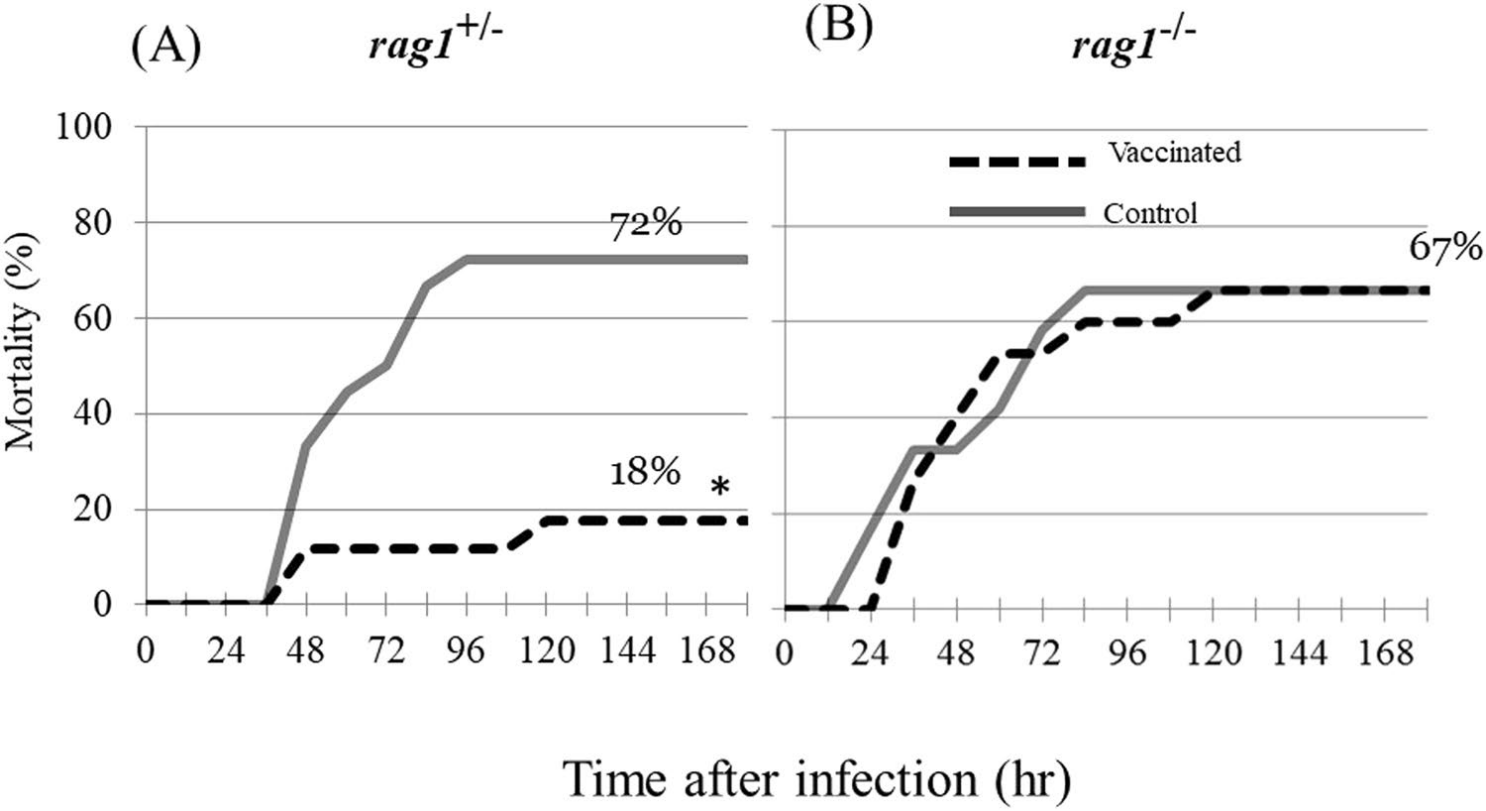
Cumulative mortality of *rag1* ^+/−^ and *rag1* ^−/−^ fish after *Vibrio* infection. The mortalities of vaccinated and unvaccinated (control) fish are shown as solid and dashed lines, respectively. The numbers in the graph indicate the final mortality rate as percentages. Asterisk (*) indicates a significant difference when compared with the control group.

Bath infection with *Vibrio anguillarum* caused symptoms of vibriosis and mortality in both strains of fish (Fig. 3). The fish began to die 12–48 h post-infection. Petechial hemorrhages were observed in on the skin and gills of dead fish in both groups (data not shown). The cumulative mortality rate of vaccinated *rag1* ^+/−^ fish was significantly lower than that of non-vaccinated fish (Fig. 3A). By contrast, no benefit of vaccination was detected in *rag1* ^−/−^ fish (Fig. 3B). These results indicated that *rag1* ^−/−^ fish did not engage adaptive immune responses against *Vibrio* infection. There was no significant difference in mortality between the strains for non-vaccinated fish, suggesting that the innate immunity of *rag1* ^−/−^ fish against *Vibrio* infection is comparable to that of *rag1* ^+/−^ fish.

### Histological observation of the thymus in rag1^+/−^ and rag1^−/−^ fish

**Figure 4:**
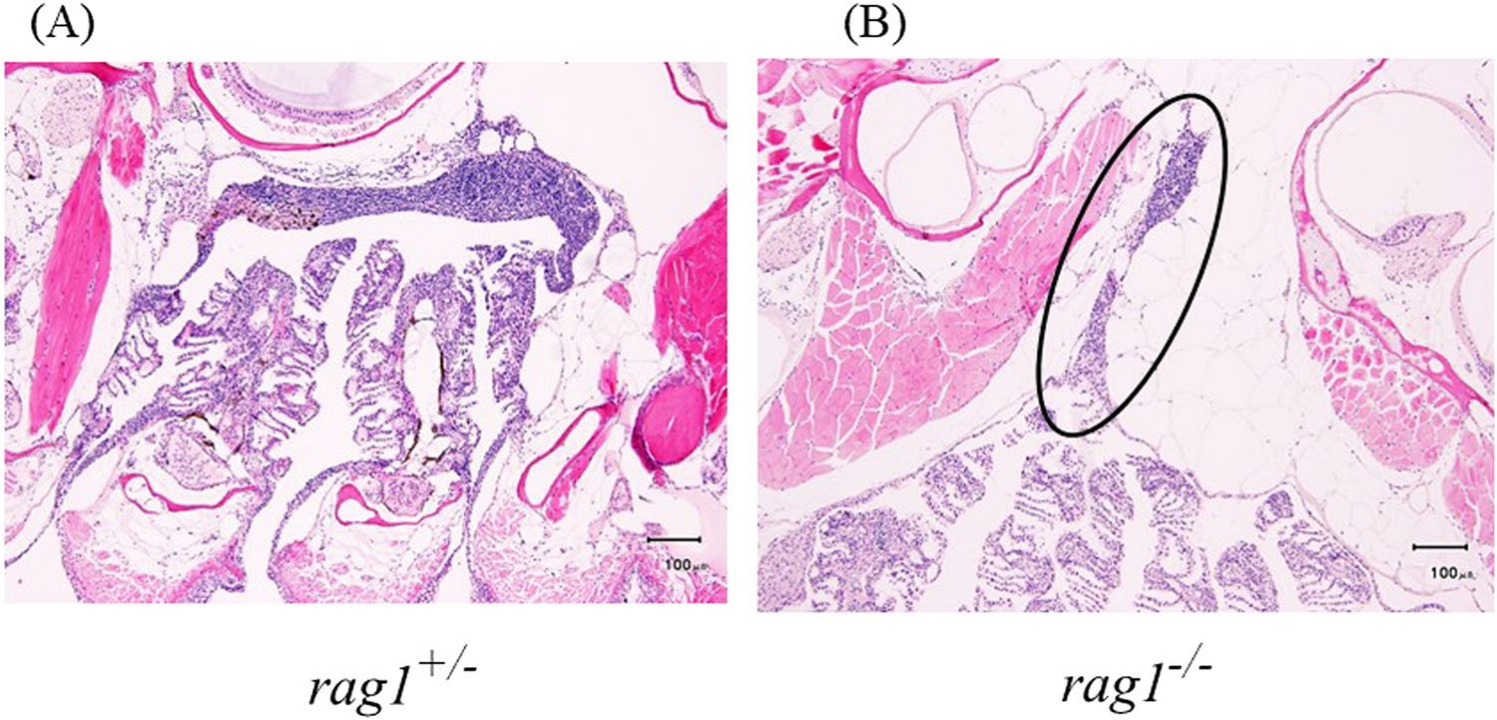
Histological observation of the hematoxylin–eosin-stained thymus in *rag1* ^+/−^ (left) and *rag1* ^−/−^ fish (right). The thymus in *rag1* ^−/−^ fish is representative of five independent samples. The thymus in *rag1* ^+/−^ fish is representative of three independent samples. A thymus was not found in two *rag1* ^+/−^ fish. The circle shows atrophic lymphocyte clump.

Histological observation revealed significant differences in thymus morphology between *rag1* ^−/−^ and *rag* ^+/−^ fish. A mature thymus, which contains a lymphocyte clump, was observed in all *rag* ^+/−^ fish (n = 5) (Fig. 4A). On the contrary, two of five *rag1* ^−/−^ fish lacked a thymus. The thymus in *rag1* ^−/−^ fish was atrophic and smaller than that in *rag1* ^+/−^ fish (Fig. 4B). Only a small lymphocyte clump was observed in the atrophic thymus. These findings illustrated that *rag1* ^−/−^ fish lack a mature thymus. No significant difference was observed in other organs between *rag1* ^−/−^ and *rag* ^+/−^ fish (data not shown).

### *In vivo* rejection of allogeneic erythrocytes

**Figure 5:**
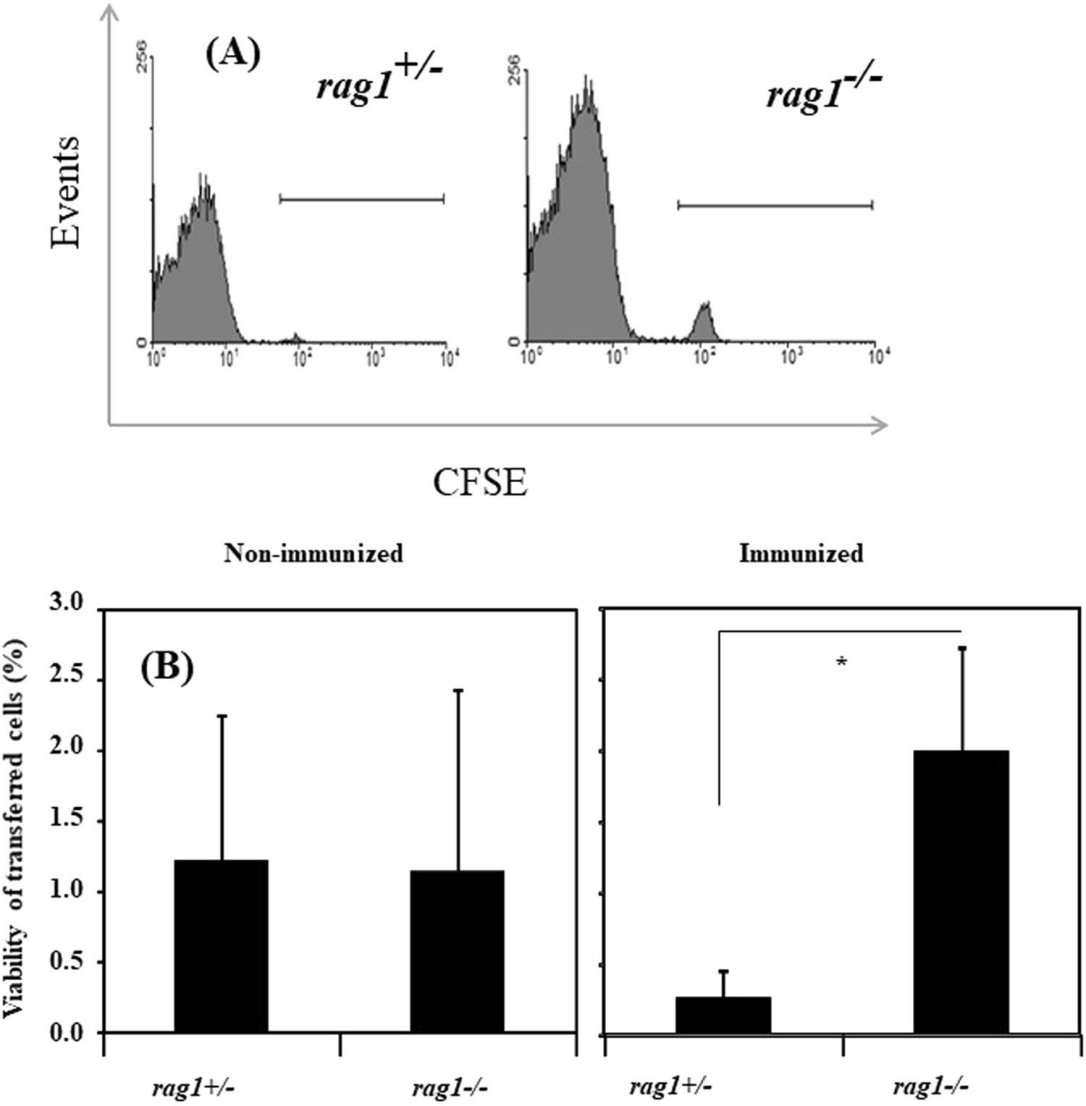
Rejection of allogeneic erythrocytes in *rag1* ^+/−^ and *rag1* ^−/−^ fish. Flow cytometry histograms show the transferred positive cells in *rag1* ^+/−^ (left) or *rag1* ^−/−^ recipients (right) (**A**). Horizontal bars in the histogram indicate the 5(6)-carboxyfluorescein diacetate *N*-succinimidyl ester-positive region. The data are representative of three independent samples. The percentage of variable transferred cells in recipients is shown in non-immunized and immunized fish (**B**). Asterisk (*) indicates a significant difference between the two strains (p < 0.05).

Donor fish, which were bled three times by pricking the caudal peduncle, could be kept alive during the immunization period. In flow cytometry, the peak of 5(6)-carboxyfluorescein diacetate *N*-succinimidyl ester (CFSE)-labeled and transferred cells was clearly detected (Fig. 5A). The percentages of transferred erythrocytes in immunized *rag1* ^−/−^ fish were significantly higher than those in immunized *rag1* ^+/−^ fish (Fig. 5B). Conversely, there was no significant difference in the survival of transferred erythrocytes in non-immunized fish between the fish groups (Fig. 5B). The results indicated that alloantigen-specific rejection was induced as a result of immunization with allogeneic cells in *rag1* ^+/−^ fish but totally impaired in *rag1* ^−/−^ fish, suggesting that *rag1* ^+/−^ fish lack alloantigen-specific CTL.

### Expression profiles of cytokines after poly(I:C) stimulation

**Figure 6:**
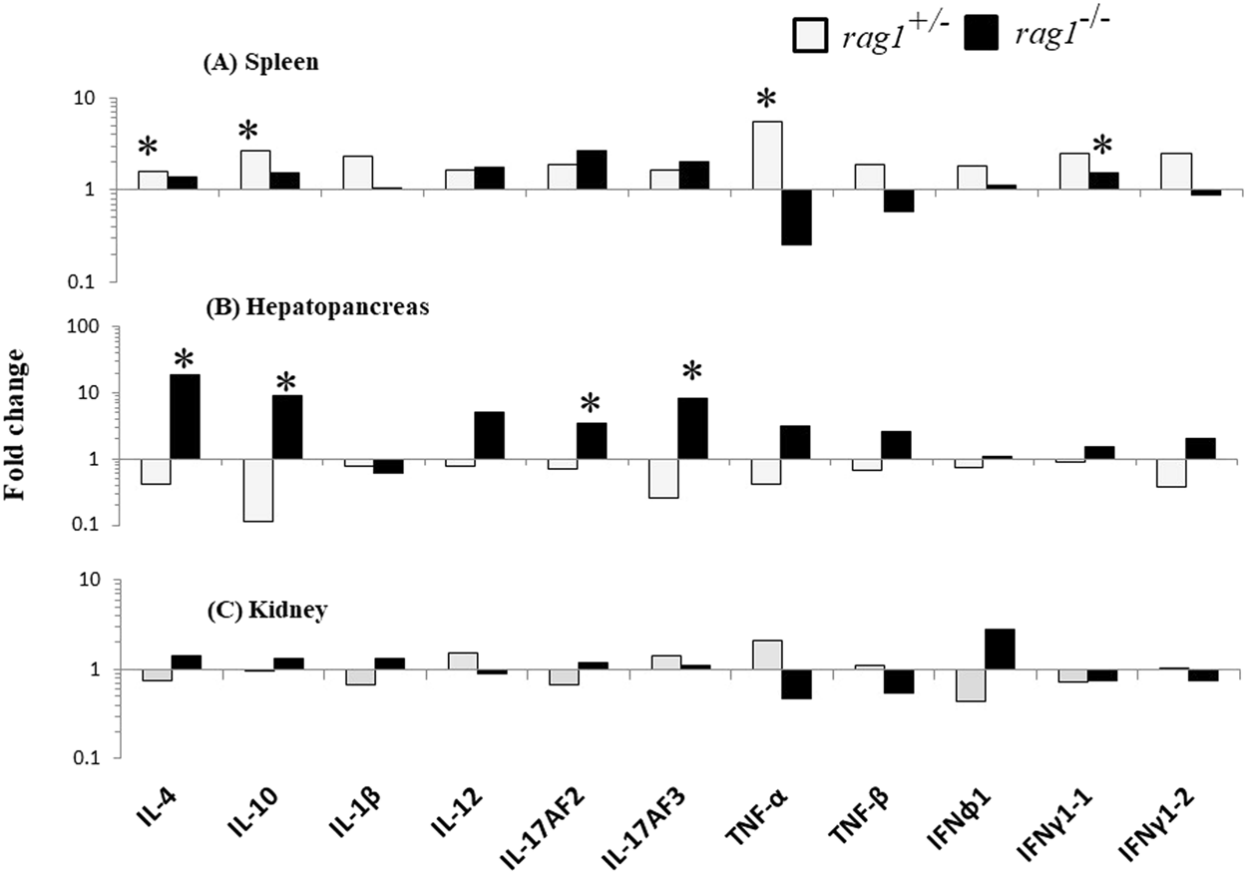
Expression of cytokines in the spleen (**A**), hepatopancreas (**B**) and kidney (**C**) of *rag1* ^+/−^ and *rag1* ^−/−^ fish. Data from the three individual experiments are shown as the mean fold change in mRNA expression relative to that in control fish. In all quantitative real-time PCR experiments, melting curve analyses were performed, and single specific melting peaks were observed, indicating amplification specificity. Statistical comparisons between stimulated and control fish were made using an unpaired *t*-test. Asterisks indicate significant differences at P < 0.05.

To reveal differences in immune homeostasis between *rag1* ^+/−^ and *rag1* ^−/−^ fish, the mRNA expression of cytokines was analyzed in the kidneys, spleen, and hepatopancreas. There was no significant difference in mRNA expression between the fish under non-stimulated conditions (data not shown). In *rag1* ^+/−^ fish, the expression of several cytokines (IL-4, IL-10, TNF-α, IFN-γ1) was significantly enhanced by poly(I:C) stimulation, whereas no enhancement was observed in the hepatopancreas. By contrast, in *rag1* ^−/−^ fish, the transcription of four cytokines (IL-4, IL-10, IL-17AF2, and IL-17AF3) was significantly increased in the hepatopancreas but not in the spleen (Fig. 6 and Fig. S2). No significant difference was observed in the kidneys. The results indicate that the production site of cytokines differs between *rag1* ^+/−^ and *rag1* ^−/−^ fish.

### Changes of phagocytic activity and cell composition after poly(I:C)-stimulation

**Figure 7:**
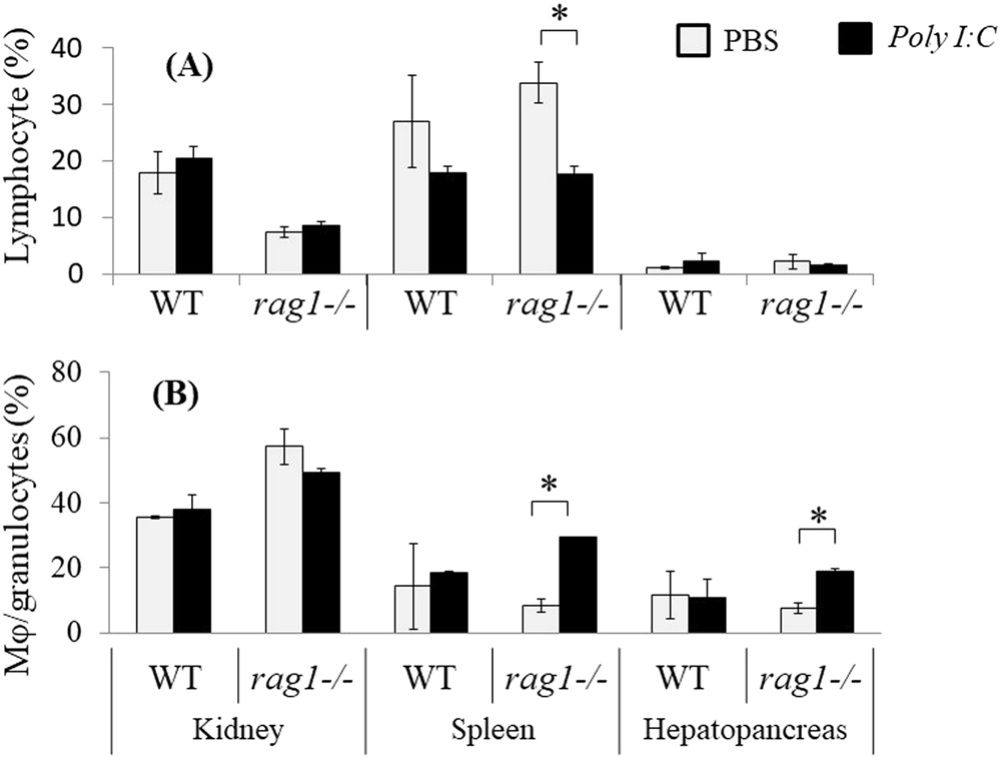
Leukocyte composition in the kidney, spleen, and hepatopancreas of wild-type (WT) (**A**) and *rag1* ^−/−^ fish (**B**) after poly(I:C) stimulation. Data from the three individual experiments are shown as the mean percentages of the lymphocyte and macrophage/neutrophil fractions. Error bars indicate standard errors. Statistical comparisons between stimulated and control fish were made using an unpaired *t*-test. Asterisks indicate significant differences at P < 0.05.

The percentage of macrophage/granulocyte was significantly increased in hepatopancreas from *rag1* ^−/−^ fish by poly(I:C)-stimulation, while there is no significant difference in hepatopancreas from *rag1* ^+/+^ fish. The representative scatter plots was shown in Fig. S3. The percentage of lymphocyte was decreased in spleen from *rag1* ^−/−^ fish after the stimulation. In contrast, the macrophage/granulocyte population was increased in the spleen (Fig. 7). Since morphological observation indicated that macrophages or granulocytes are dominant cells in large cell population from hepatopancreas or from kidney, respectively, suggesting that macrophage/granulocyte fraction mainly consists of macrophages in hepatopancreas, and granulocytes in kidney (data not shown).

## Discussion

*Rag*-null mice and humans develop SCID as a result of T- and B-cell deficiency and thus require pathogen-free environments for survival^21, 22^. This phenomenon is evidence that the clonal diversity of T and B cells is essential for maintaining the life of higher vertebrates. By contrast, *rag1*-null fish can survive under non-SPF conditions for at least for 2 years, and they exhibited immunological memory-like responses to intracellular bacterial infection^17^. These findings raise fundamental questions regarding whether *rag1*-null zebrafish actually lack T- and B-cell function. If this is true, then it can be hypothesized that teleosts intrinsically rely more heavily on Rag-independent immunity than mammals.

Although previous studies suggested that *rag1*-deficient fish lack the ability to generate antibody diversity at a genetic aspect^16, 20^, there was no direct evidence that they lack immunoglobulins and functional B-cells. As zebrafish are not of suitable size for collecting sufficient amounts of blood, it was difficult to detect the target proteins in their organs or blood. To overcome this disadvantage, we pooled blood from several fish and concentrated proteins of similar molecular weights as IgM via gel filtration chromatography. Highly sensitive chemiluminescent Western blotting did not detect IgM in serum from *rag1* ^−/−^ fish, suggesting that they do not produce IgMs including natural antibodies. In addition, agglutinating antibody was also undetectable in *rag1* ^−/−^ fish after repeated immunization with a *Vibrio* vaccine, suggesting that they cannot develop a functional antibody response. A previous report described that *rag1* ^−/−^ fish had a smaller lymphocyte population than *rag1* ^+/−^ fish based on flow cytometry scattergrams of peripheral and kidney leukocytes^20^. This was confirmed by the present findings that *rag1* ^−/−^ fish do not possess IgM-positive lymphocytes. Although the remote possibility that IgZ-positive cells exist in *rag1* ^−/−^ fish remains, we concluded that *rag1* ^−/−^ fish lack mature B-cells and cannot produce immunoglobulins.

It is known that crosstalk between thymocytes and thymic epithelial cells preserves the thymic architecture, and in fact, several types of Rag mutations result in defective thymic stromal maturation in mouse models^3^. Although previous work observed no remarkable abnormalities of the intestine, kidneys, and liver in *rag1* ^−/−^ fish, no information on thymus development has been reported in the null animal^23^. In the present study, we clearly observed atrophy of the thymus in *rag1* ^−/−^ fish. These results infer that intercellular communication malfunction similar to that in mammals occurred in the *rag1* ^−/−^ zebrafish thymus, thereby suggesting evolutionary conservation of the mechanism of T-cell maturation and education in the thymus.

Allograft rejection experiments represent a typical assay for evaluating antigen-specific and MHC-restricted cellular immunity. Several studies revealed that the alloantigen-specific immune response is executed by CTL in teleost fish^4, 5, 24, 25^. In fish, grafting of allogeneic skin or scales has been employed as a simple test for examining allograft rejection by the cellular immune response, but the methodology is not easily applied to a wide variety of fish species, especially small fish such as zebrafish. *In vivo* rejection utilizing erythrocytes as an allograft has previously been demonstrated in rainbow trout, suggesting that CTL recognized MHC class I molecules as a major determinant for rejecting allogeneic erythrocytes^26^. Thus, we considered that allogeneic erythrocyte rejection is a useful modality for evaluating CTL activity *in vivo*, and we attempted to apply this assay to zebrafish, a small fish species. Immunized *rag1* ^+/−^ recipients rejected allogeneic erythrocytes from donor fish, whereas *rag1* ^−/−^ fish could not reject donor cells. This finding suggests that *rag1* ^−/−^ fish lack CTL activity.

Horn and Hanson (2012) reported that re-infection with the intracellular bacterium *Edwardsiella ictaluri* provides *rag1* ^−/−^ fish immunological memory-like protection, suggesting that NK-like cells might play an important role as innate memory cells^17^. By contrast, the present study did not identify any memory-enhancing effect of vaccination in *rag1* ^−/−^ fish against *Vibrio anguillarum* challenge. The contradiction between these findings is presumably attributed to differences in the intracellular and extracellular bacteria. Because NK-like cells or innate lymphoid cells (ILCs) may not efficiently eliminate extracellular bacteria such as *Vibrio anguillarum* and a specific antibody was not secreted in *rag1* ^−/−^ fish, it appears that they did not receive any benefit from vaccination against *Vibrio* infection. It is noteworthy that no difference in mortality was observed between non-vaccinated *rag1* ^−/−^ and *rag1* ^+/−^ fish, inferring that *rag1* ^−/−^ fish possess protective ability equivalent to that of *rag1* ^+/−^ fish without any significant contribution from T and B cells to host defense.

The present study uncovered a significant difference between adaptive immunity-deficient and normal fish. Although the spleen is a secondary lymphoid organ harboring functional T- and B-cells in vertebrates, cytokine mRNA expression was not increased after poly(I:C) stimulation in the spleen from *rag1* ^−/−^ fish. On the contrary, the hepatopancreas is inferred to be an important site of cytokine production in adaptive immunity-lacking fish. Although absolute level of the cytokines expression in hepatopancreas was lower than that in spleen (Fig. S2), significant upregulation of the cytokines in *rag1* ^−/−^ was detected in only hepatopancreas by poly(I:C) stimulation. This finding suggests that hepatopancreas compensates the roles of secondary immune organs by lacking adaptive immunity. In mammals, the primary site of ILC development is the liver in the fetus, and liver-resident NK cells play important roles in immune homeostasis^27–29^. Flow cytometry revealed that the numbers of macrophages in the hepatopancreas in *rag1* ^−/−^ fish were increased by stimulation, whereas they were not increased in wild-type fish. Thus, macrophage in primitive vertebrates, but not ILC, may compensate for the functions of T and B cells in the liver or hepatopancreas.

The zebrafish is widely employed in immunological and hematological studies as a vertebrate model, contributing to both fish immunology and human medical science^22, 30–38^. The present and preceding studies illustrated that teleosts rely more heavily on rag-independent immunity than mammals and suggest that zebrafish can serve as a better animal model for exploring the potential of innate immunity than mice. At present, it remains unclear which factors compensate, at least in part, to enable the long-term survival of fish under non-SPF rearing conditions. Jima *et al*. (2009) reported that the expression of genes encoding components of the complement and coagulation systems was enhanced in *rag1* ^−/−^ fish^23^. However, we did not detect significant differences in the serum concentration of complement component C3 protein between *rag1* ^−/−^ and *rag1* ^+/−^ fish (data not shown). In addition, although we did not find *rag1* ^−/−^ fish-specific upregulation of several cytokine mRNA under normal (uninfected) conditions, poly(I:C) stimulation induced the differential expression of cytokine mRNAs between *rag1* ^−/−^ and *rag1* ^+/−^ fish. Thus, enhanced immune factors that could support the innate defense of *rag1* ^−/−^ fish independently from B- and T-cell–mediated adaptive immunity should be investigated for various pathogens with different infection routes. These *rag1* ^−/−^ fish appear to possess more efficient innate immune defenses than mammals, and thus, they may provide insight into a novel methodology to enhance the innate defense of immune-compromised mammals, such as patients with terminal cancer and other adaptive immune deficiencies.

## Materials and Methods

### Fish

The *rag1* ^−/−^ line (rag1^t26683^) was previously generated at the Hubrecht Institute^16^ and provided by the Tübingen 2000 Screen Consortium. *rag1* ^−/−^ fish were reproduced at the National Research Institute of Aquaculture and Kyushu University. *rag1* ^+/−^ fish were generated by crossing the null strain with the normal wild-type or albino strain. They were genotyped using previously described PCR methods^16^. Adult fish (0.1–0.5 g, 5 weeks–6 month old) were used in the experiments. *rag1* ^+/−^ or wild-type (*rag1* ^+/+^) fish were used as controls. All animal experiments were performed in accordance with the guidelines of the Animal Experiments Committee at Kyushu University, and all experimental protocols were endorsed by the committee.

### Gel filtration, SDS-PAGE, and Western blotting

Fish (wild-type and *rag1* ^−/−^) were anesthetized in 20 ppm quinaldine and bled by caudal amputation into a hematocrit capillary tube. The blood was allowed to clot at 4 °C for 2 h and centrifuged at 3000 rpm for 5 min. The tubes were cut at the boundary between serum and blood cells to collect serum, which was cleared by centrifugation at 12,000 rpm for 10 min. Nine microliters of pooled serum were diluted in 180 μl of 20 mM sodium phosphate buffer containing 0.9% NaCl (pH 7.4). The diluted serum was passed through a Superdex 200 gel filtration column (1 × 30 cm) equilibrated with same buffer and eluted at 0.5 ml/min. The eluted protein was precipitated with 17% (w/v) trichloric acid, washed with acetone, and dissolved in a 1/10 volume of SDS sample buffer containing 5% 2-mercaptoethanol.

Protein samples from the fractions (#1–5 in Fig. 1A) were subjected to SDS-PAGE on 10% gel under reducing conditions. For Western blotting, the separated proteins were electroblotted onto a nitrocellulose membrane (Hybond-C Extra, GE Healthcare Life Sciences, Little Chalfont, UK) in a transfer buffer composed of 100 mM Tris, 192 mM glycine, 20% methanol, and 0.02% SDS. After blocking with 5% skim milk in PBS (SM-PBS), the membrane was treated with anti-ayu (*Plecoglossus altivelis*) IgM rabbit serum (a gift from Dr. Masakazu Kondo, National Fisheries University, Shimonoseki, Japan; 1/200 diluted in SM-PBS) and then with peroxidase-conjugated goat anti-rabbit IgG (diluted 1/2,000 in SM-PBS; Cappel, MP Biomedicals, Santa Ana, CA, USA). This primary antibody was selected from three candidates of anti-teleost IgM antibodies by Western blotting (data not shown). Between each step, the membrane was washed gently with 0.05% Tween-PBS. Chemiluminescent detection was performed using ECL Western Blotting Detection Reagents (Amersham, GE Healthcare BioScience Corporate, Piscataway, NJ, USA) followed by exposure to Fuji UR X-ray film, which was developed with GBX Development and Fixation solutions (Kodak Co., Rochester, NY, USA).

### Agglutinating antibody titer

A commercial *Vibrio* vaccine (Kyoritsu Seiyaku co., Ltd. Tokyo, Japan) and an emulsion of ISA 763 A (Seppic, France) were mixed at a 1:3 ratio. *rag1* ^+/−^ and *rag1* ^−/−^ fish (6 fish per strain) were injected with 10 µl of the mixture. A second vaccination was performed 2 weeks after the primary vaccination. The vaccinated fish were bled by caudal amputation using a heparinized hematocrit capillary. The collected blood was centrifuged at 11,000 rpm for 3 min, and 4.0–5.0 µl of plasma were obtained. The plasmas were serially diluted with PBS in a U-bottom microtiter plate (initial dilution 1:8). Ten microliters of the vaccine suspension in PBS were added in to well containing 10 µL of the plasma dilution. The plate was mixed for 15 min at room temperature and centrifuged at 1000 rpm. Agglutination was observed, and the antibody titers were scored as the highest dilution giving positive agglutination.

### Histological observation of organs

At five weeks post-hatching, both strains of fish were anesthetized and dissected, and tissue surrounding the thymus, brain, muscle, spine, kidneys, and hepatopancreas was isolated. The organs were fixed in Davidson’s solution for 24 h. The fixed organs were dehydrated through an ethanol series, treated with xylene, and embedded in paraffin wax. The paraffin-embedded tissues were sectioned at 3 μm and stained with hematoxylin–eosin. The tissue section was observed by light microscopy.

### Flow cytometry of IgM-positive cells

Fish were anesthetized and bled using heparinized hematocrit capillaries as described previously to separate peripheral blood cells, and then they were dissected to isolate the trunk kidney. The kidney cells were isolated by pressing the tissues through a 150-gauge mesh stainless steel sieve in RPMI-1640 medium (Nissui Pharmaceutical, Tokyo, Japan). The dispersed cells were treated with anti-ayu IgM rabbit serum (diluted 1/500) and then with FITC-conjugated goat anti-rabbit IgG (diluted 1/50; Cappel, MP Biomedicals) for 40 min on ice. Normal rabbit serum was used as a negative control. Between each step, the cells were washed with RPMI-1640 by centrifuging at 300 × *g*. IgM^+^ cells in the lymphocyte gate were detected by flow cytometry (EpicsXL, Beckman Coulter).

### Protective effect of vaccination against Vibrio anguillarum infection

It is known that *Vibrio anguillarum* is useful for bacterial infection model on zebrafish^39^, and vaccine against *V*. *anguillarum* are available in many fish species^40^. *rag1* ^+/−^ (n = 17) and *rag1* ^−/−^ (n = 15) zebrafish were vaccinated with formalin-killed *V*. *anguillarum* VR775 by intraperitoneal injection (1.0 × 10^6^ cells/5 μl of PBS per fish fish). The second and third immunizations were performed at 7-day intervals. Control fish (*rag1* ^+/*−*^, n = 18; and *rag1* ^−/−^, n = 12) were injected with the same volume of PBS. At two weeks after the final vaccination and PBS-injection, the vaccinated and control fish were challenged by immersion with the same strain of *V*. *anguillarum* (1.0 × 10^7^ cells/ml) in 200-ml beakers for 1.5 h at 28 °C. Subsequently, they were transferred to 2-l tanks (5–8 fish/tank) and kept at 28 °C. The number of dead fish was recorded daily for 7 days. The water in each tank was changed every 2 days.

### *In vivo* rejection of transferred erythrocytes

To evaluate adaptive cell-mediated immunity, we established *in vivo* allograft rejection experiments in zebrafish (Fig. S1) in which *rag1* ^+/−^ and *rag1* ^−/−^ fish were employed as recipients, and wild-type fish (*rag1* ^+/+^) served as donor fish. Donors were anesthetized and pricked with a 27-gauge needle just ventral to the lateral line of the caudal peduncle. The wound with a few microliters of bleeding was dipped in a Petri dish with D-MEM (Invitrogen) containing 1% heparin, and the blood cells were then suspended in the medium. The suspension was centrifuged at 300 × *g* for 5 min, and erythrocytes were collected for use as allogeneic immunogens. The donors could be kept alive and maintained during the experiment for repeated blood cell collection. The recipient fish (weighting approximately 0.3 g) were intraperitoneally injected with the donor erythrocytes (1 × 10^5^ cells in 10 μl of PBS/fish). The second and third immunizations were performed with the same doses of cells freshly prepared from the same donor at 10-day intervals. At 10 days after the final immunization, the recipient fish were bled from the tail using the previously described method. The blood was suspended in D-MEM, and the erythrocytes were precipitated by centrifugation at 300 × *g* for 5 min. The erythrocytes were incubated with 0.3 μg/ml CFSE (Sigma-Aldrich) for 20 min at 25 °C and washed twice in D-MEM. The recipient fish received 1 × 10^6^ of the CFSE-labeled erythrocytes from the same donor. The erythrocytes from the recipients were harvested at 4 days post-transplantation by the aforementioned method, and the percentage of surviving CFSE-positive cells was measured by flow cytometry. Three sets of donors and recipients were tested. In addition, allograft rejection by non-immunized fish was investigated using the same procedure.

To confirm allogenecity between donors and recipients, the genotypes of the donor/recipient pairs were investigated by PCR using primers for the U-lineage of MHC class I^41^. All pairs exhibited different genotypes of the U-lineage (data not shown).

### Expression analysis of cytokines by real-time quantitative PCR

The fish were intraperitoneally injected with 5 μl of 0.8 μg/μl poly(I:C) solution and sampled 24 h post-stimulation. The fish were anesthetized and dissected to isolate the trunk kidney, spleen, and hepatopancreas. Control fish were injected with the same amount of PBS. Total RNA was extracted from these organs using ISOGEN Reagent (Nippon Gene, Tokyo). First-strand cDNA was synthesized from total RNA using Moloney murine leukemia virus reverse transcriptase (Invitrogen, Life Technologies, Carlsbad, CA, USA) with an oligo (dT) primer according to the manufacturer’s instructions. Eleven cytokines related to inflammation and anti-inflammation (IL-1β, IL-17AF2, IL-17AF3, TNFα-1, TNFα-2, IFNφ1, IFNγ1-1, IFNγ1-2, IL-10, and IL-4) were selected in this study. The primers used for real-time PCR are listed in Table S1. The internal control for normalization was EF-1α. The sequences or primer sets of IFN-γ, perforin, and EF-1α are indicated in the reference articles. Quantitative real-time PCR was performed in duplicates using an Mx 3000 P System (Stratagene, La Jolla, CA, USA) in 16-µl reaction mixtures containing 2 μl of template cDNA, 0.5 μM primers, and other reagent components from the Fast Start DNA Master SYBR Green (Roche Applied Science, Mannheim, Germany). Thermal cycling was performed using a two- or three-step thermal cycling mode composed of initial denaturation for 1 min at 95 °C followed by 40 cycles of 10 s at 95 °C and 30 s at 60 °C (IL-1, IL-4, IL-10, IL-17AF3, TNFα1, IFNγ1-1, IFNγ1-2, IFNφ1, and EF-1α) or 40 cycles of 10 s at 95 °C, 15 s at 60 °C, and 30 s at 72 °C (IL-12, IL-17AF2, TNFα2). The relative quantitative value of each gene was calculated according to the standard curve from a serial dilution of a reference cDNA in the same plates and normalized by the level of EF1α.

### Leukocyte composition assay via flow cytometry

The fish were injected with poly(I:C) and dissected to isolate the trunk kidney, spleen, and hepatopancreas as described previously. The cells were isolated by pressing the tissues through a 150-gauge mesh stainless steel sieve in RPMI-1640 medium (Nissui Pharmaceutical). The erythrocytes were lysed with erythrocyte lysis buffer (0.15 M NH_4_Cl, 1.0 mM KHCO_3_, 0.1 mM EDTA), and the leukocytes were washed twice with PBS.

3,3-Dihexyloxacarbocyanine (DiOC_6_, Molecular Probes, Eugene, OR, USA) staining was used to enhance erythrocyte fluorescence and side scatter according to a previously described method for fish^42^. The isolated cells were treated with DiOC_6_ (10 μg/ml), incubated for 10 min at room temperature, and washed twice with PBS. The lymphocyte and macrophage/granulocytes fractions were gated according to the methods for zebrafish^20^.

## Electronic supplementary material

Supplementary Figures
