# Supplementary material for: Comprehensive validation of T- and B-cell deficiency in rag1-null zebrafish: Implication for the robust innate defense mechanisms of teleosts
Source: Sci Rep. 2017 Aug 8;7:7536. doi: 10.1038/s41598-017-08000-2 (PMC5548773; doi:10.1038/s41598-017-08000-2)
Supplement: Supplementary file 1 — Supplementary Figures [file 41598_2017_8000_MOESM1_ESM.pdf]

Comprehensive validation of T- and B-cell deficiency in *rag1*-null zebrafish: Implication for the robust innate defense mechanisms of teleosts

Yumie Tokunaga[1,4], Masamichi Shirouzu[1], Ryota Sugahara[1], Yasutoshi Yoshiura[2], Ikunari Kiryu[3], Mitsuru Ototake[3], Takahiro Nagasawa[1], Tomonori Somamoto[1]*, Miki Nakao[1].

1. Laboratory of Marine Biochemistry, Department of Bioscience and Biotechnology, Graduate School of Bioresource and Bioenvironmental Sciences, Kyushu University, Fukuoka 812-8581,

2. Yashima station, Stock Enhancement and Management Department, National Research Institute of Fisheries and Enhancement of Inland Sea, Japan Fisheries Research and Education Agency, 243 Yashima-higashi, Takamatsu, Kagawa, 761-0111, Japan.

3. National Research Institute of Aquaculture, Fisheries Research Agency, Tamaki, Mie, 519-0423, Japan.

4. Instituto de Medicina Molecular, Faculdade de Medicina, Universidade de Lisboa, Lisboa, Portugal.

***Corresponding author:**

Tomonori Somamoto

Phone: (81)-92-642-2895; Fax: (81)-92-642-2897

E-mail: somamoto@agr.kyushu-u.ac.jp

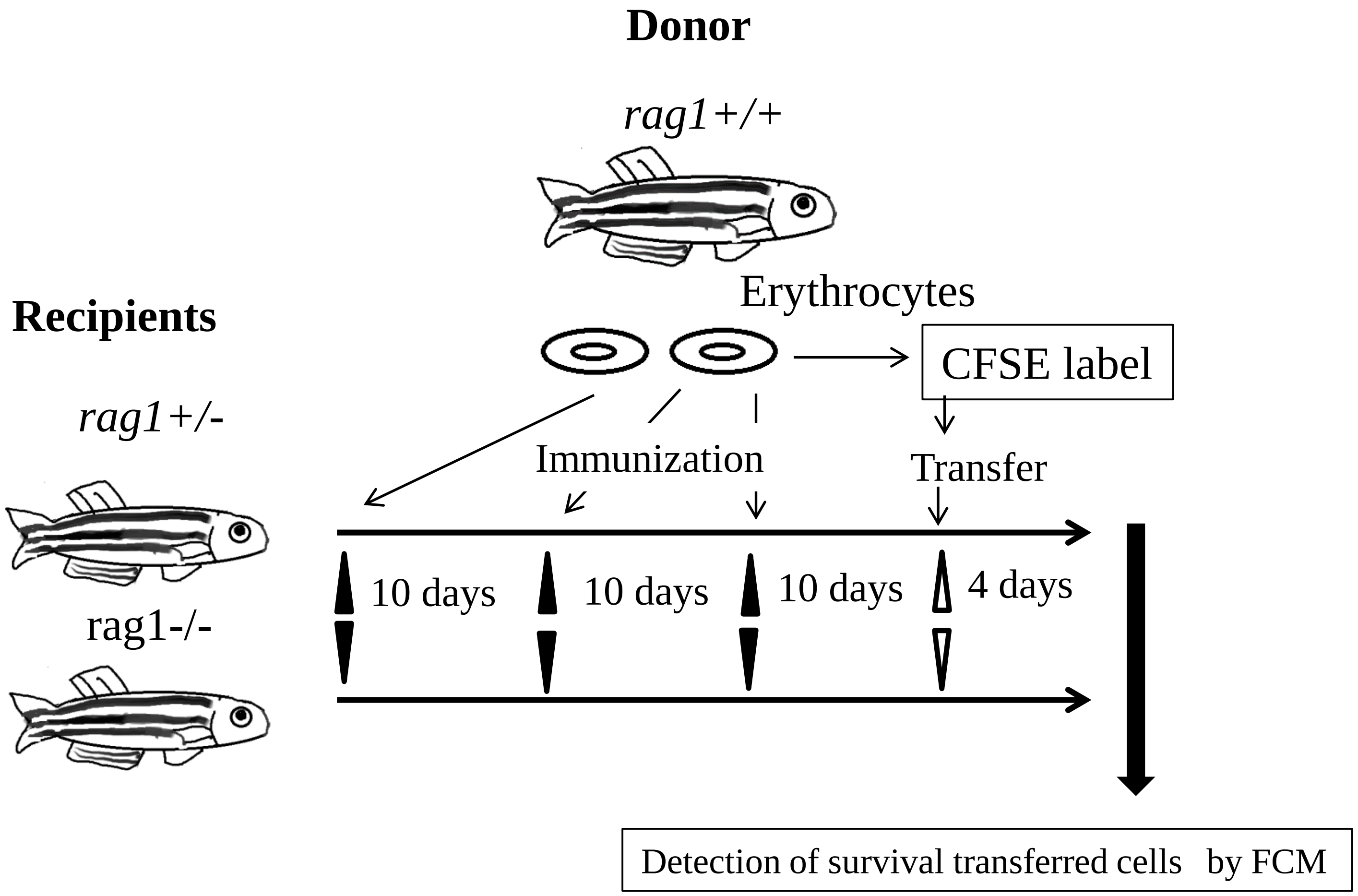

Fig. S1. Experimental scheme for the rejection of in vivo allogeneic erythrocytes in zebrafish. Black arrowheads indicate immunization with 1.0 × 10^6 allogeneic erythrocytes. Open arrowheads indicated transplantation with 1.0 × 10^7 5(6)-carboxyfluorescein diacetate *N*-succinimidyl ester (CFSE)-labeled allogeneic erythrocytes. Arrow indicates the collection of erythrocytes from the recipient and detection of CFSE-labeled erythrocytes by flow cytometry. Three pairs of donors and recipients were tested in each strain of fish.

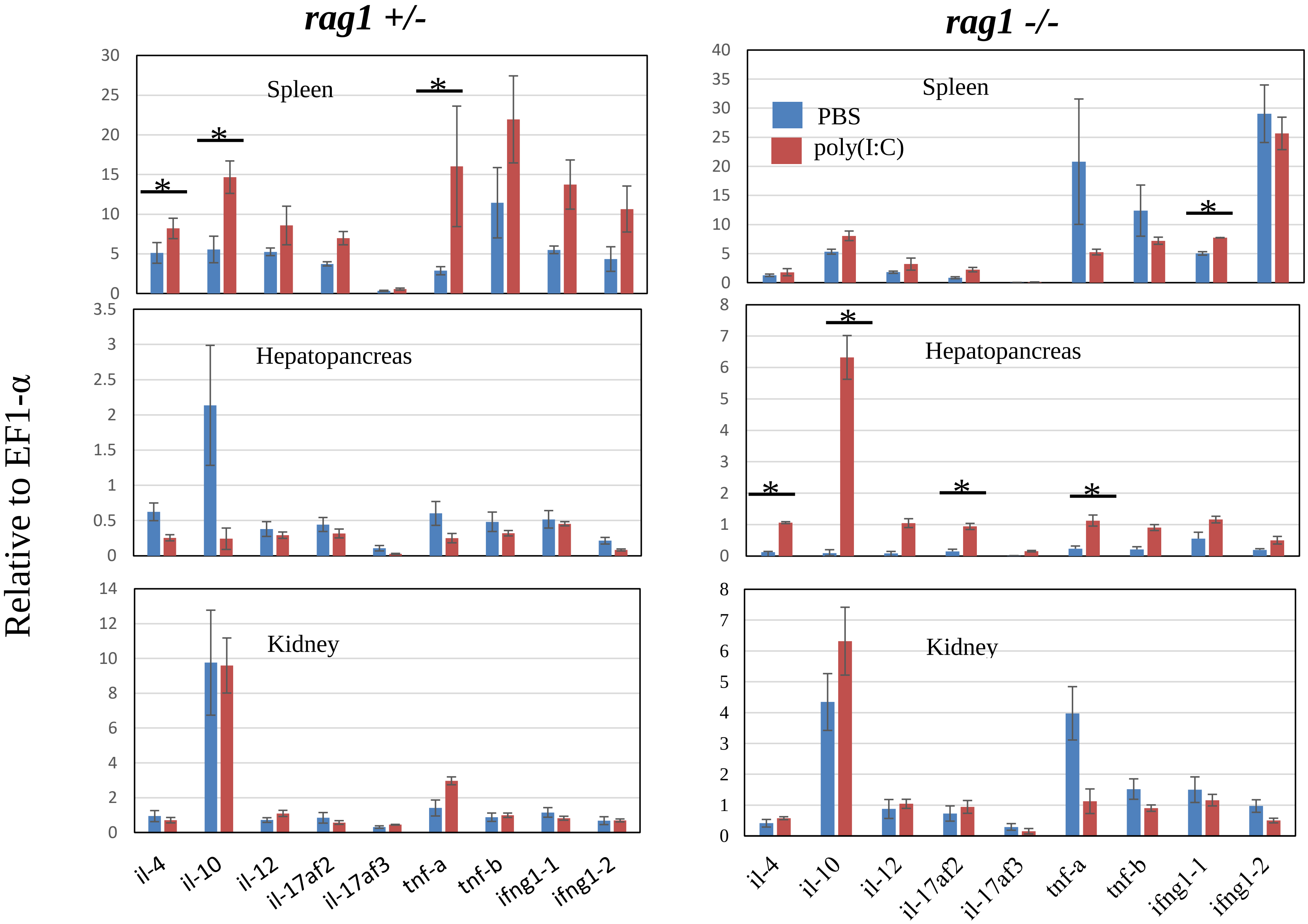

Fig. S2. Expression of cytokines in the spleen, hepatopancreas and kidney from *rag1*$^{+/-}$ and *rag1*$^{-/-}$ fish that were injected with PBS (■) and poly(I:C) (■). Data from the three individual experiments are shown as the mean of relative value to EF1-α. Statistical comparisons between stimulated and control fish were made using an unpaired *t*-test. Asterisks indicate significant differences at P < 0.05.

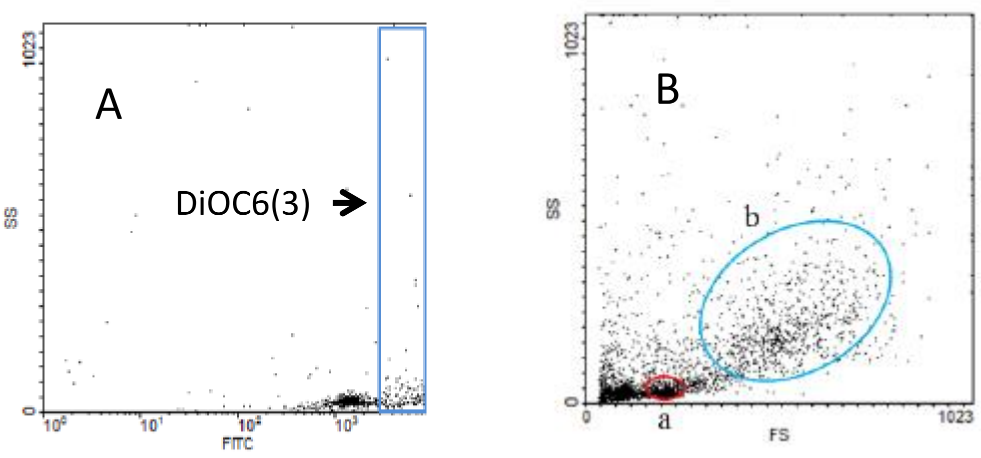

Fig. S3. Representative scatter plots of kidney leukocytes from wildtype zebrafish in flow cytometry analysis. The blue rectangle gate indicates DiOC6(3)-positive cells (A). Forward scatter (FS) and side scatter (SS) analysis: Lymphocyte (a) and macrophage/neutrophil (b) gate (B).

Table S1 Primers for real time PCR.

Target genes	Primer sequences (5'→3')
IL-1β	F: TTCCCCAAGTGCTGCTTATT
	R: AAGTTAAAACCGCTGGTCA
IL-4	F: AGTCACGCTGCTGATGAAGA
	R: AACTTGGTCTTGGGCTTTTT
IL-10	F: ATAGGATGTTGCTGGGTTGG
	R: GTGGATGAAGTCCATTTGTGC
IL-12	F: GCTGAAGGAGTGTTTCCTCAGT
	R: TGACATCATTTCCTGTGCTCTC
IL-17AF2	F: CTGCACTGGGCTTCAAAGAT
	R: TCAATCTGAGGACGGAAAGG
IL-17AF3	F: TCTCTGTCGCCTTGGACATAC
	R: GTCCTCCTCACCGTCTTTTG
TNFα1	F: GCGCTTTTCTGAATCCTACG
	R: TGCCCAGTCTGTCTCCTTCT
TNFα2	F: AAGCCAAACGAAGAAGGTCA
	R: AACCCATTTCAGCGATTGTC
IFNφ1	F: ACGACAGAATCTCTGAACCT
	R: GTCAGGACTAAAAACTTCAC
IFNγ1-1	F: TTCAGACAACCAGCGCATAC
	R: AACCCAATCCTTTGCAAGC
IFNγ1-2	F: TGCAGAGCTCAGGACGTATG
	R: CTTTAGCCTGCCGTCTCTTG
EF1α	F: AACAGCTGATCGTTGGAGTCAA
	R: TTGATGTATGCGCTGACTTCCT
